# Successful management of an atrio-esophageal fistula after atrial fibrillation ablation: a case report

**DOI:** 10.1186/s44215-024-00136-8

**Published:** 2024-02-21

**Authors:** Kohei Hachiro, Noriyuki Takashima, Kentaro Matsuoka, Katsushi Takebayashi, Sachiko Kaida, Masaji Tani, Tomoaki Suzuki

**Affiliations:** 1 Division of Cardiovascular Surgery, Department of Surgery, Shiga University of Medical Science, Setatsukinowa-cho, Otsu, Shiga 520-2192 Japan; 2 Department of Surgery, Shiga University of Medical Science, Setatsukinowa-cho, Otsu, Shiga 520-2192 Japan

**Keywords:** Atrio-esophageal fistula, Right posterolateral thoracotomy, Cardiopulmonary bypass

## Abstract

**Background:**

Atrio-esophageal fistula is a rare but still a catastrophic complication of radiofrequency ablation of atrial fibrillation. We report a successful case of atrio-esophageal fistula with right posterolateral thoracotomy and right femoral cannulation of cardiopulmonary bypass.

**Case presentation:**

A 67-year-old man underwent radiofrequency ablation for atrial fibrillation. Nineteen days later, he developed cerebral infarction, and computed tomography showed air in the left atrium. He was transferred to our hospital for surgery. The upper body was placed in the left lateral decubitus position, and the lower body was placed in the left hemilateral decubitus position. The surgical approach was a right posterolateral thoracotomy in the 5th intercostal space. At first, the esophagus was transected at the diaphragm and tracheal carina levels. Then, an arterial line was inserted into the right common femoral artery and venous line into the right common femoral vein. Three U-shaped sutures of 3-0 polypropylene were placed to stop bleeding from the atrium. The esophagus was removed while snaring the 3-0 polypropylene sutures. There were two holes in the esophagus. Four drains were placed to clean the repaired site. After chest closure, the patient was placed supine. Cervical esophagostomy and enterostomy were performed. Gastric tube reconstruction via the retrosternal route was performed on postoperative day 28, and the patient was transferred to another hospital for rehabilitation on postoperative day 99.

**Conclusions:**

It is important to thoroughly discuss with esophageal surgeon how to reach the heart and esophagus, and how to reconstruct the esophagus later.

## Background

Atrio-esophageal fistula (AEF) is a rare but still a catastrophic complication of radiofrequency ablation of atrial fibrillation. It is reported to occur in 0.1–0.25% of atrial fibrillation ablation [1], and the mortality rate has been reported to be around 70% [2]. However, optimal treatment strategy remains unclear. We report a successful case of AEF with right posterolateral thoracotomy and right femoral cannulation of cardiopulmonary bypass.

## Case presentation

**Fig. 1:**
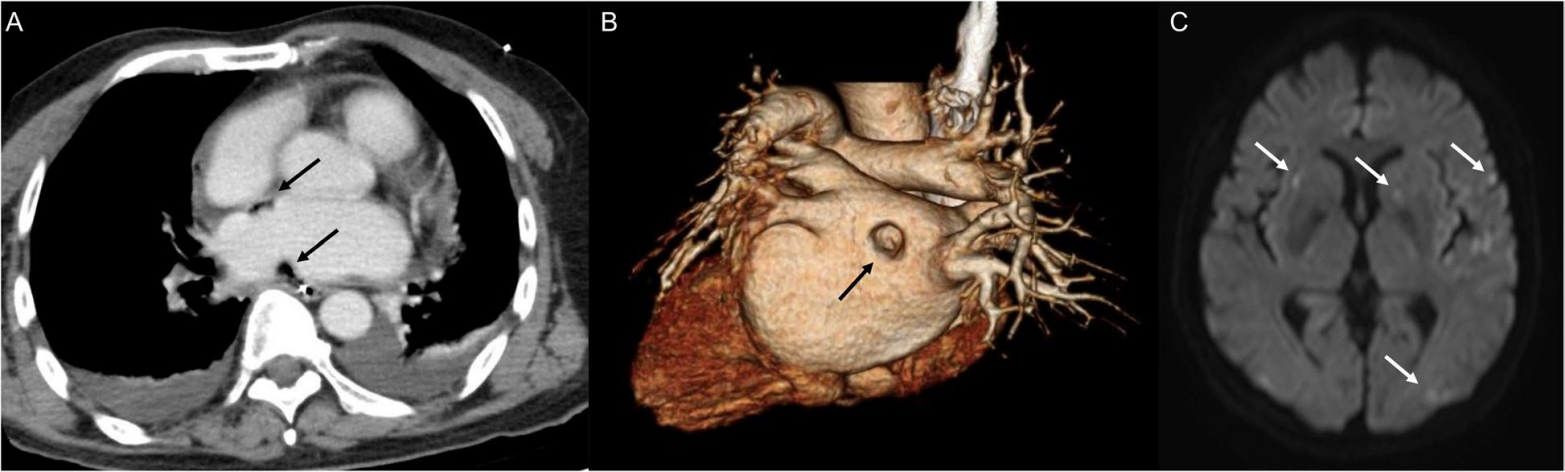
Preoperative enhanced computed tomography showed the presence of air in the left atrium (**A**). 3D image showed contrast defect in the posterior wall of the left atrium (**B**). Magnetic resonance imaging showed multiple cerebral infarction (**C**)

A 67-year-old man underwent radiofrequency ablation for atrial fibrillation including Box isolation while his esophageal temperature was monitored. Nineteen days later, he developed left paresis. Computed tomography showed air in the left atrium (Fig. 1A) and 3D image showed contrast defect in the posterior wall of the left atrium (Fig. 1B). Magnetic resonance imaging showed multiple cerebral infarctions (Fig. 1C). He was transferred to our hospital for surgery. After discussion with an esophageal surgeon, we decided to perform emergency left atrium repair and removal of the esophagus at the same time. Assuming difficulty in controlling bleeding and stabilizing hemodynamics, we planned to perform cardiopulmonary bypass as mechanical circulatory support.

**Fig. 2:**
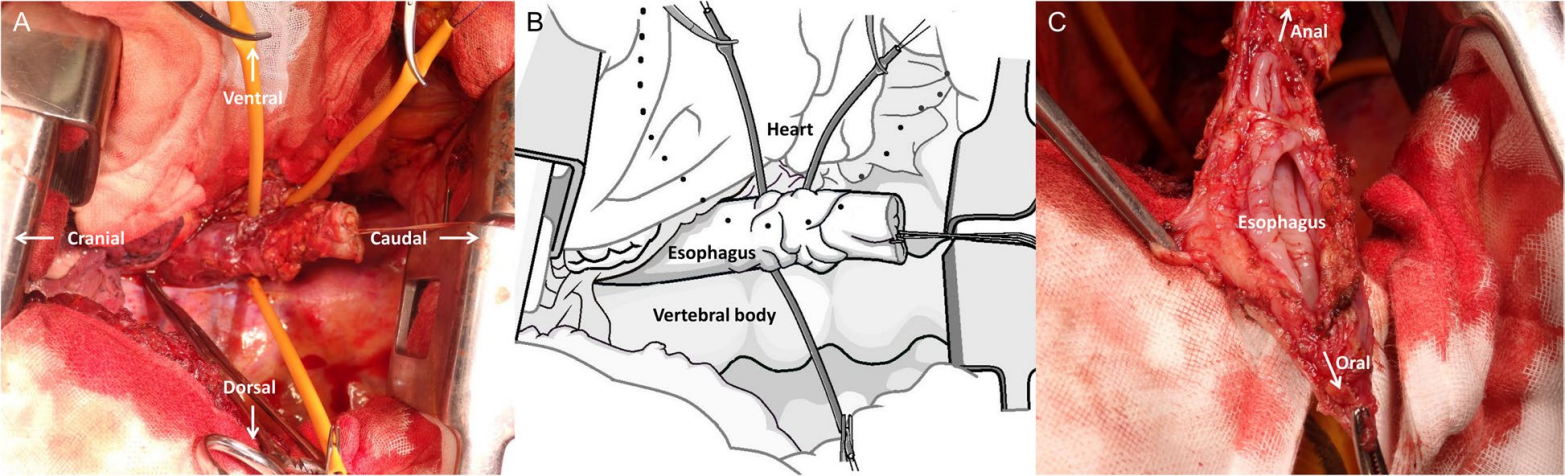
Three pairs of U-shaped sutures with 3-0 polypropylene to control bleeding (**A**). Schema (**B**). Removed esophagus (**B**)

**Fig. 3:**
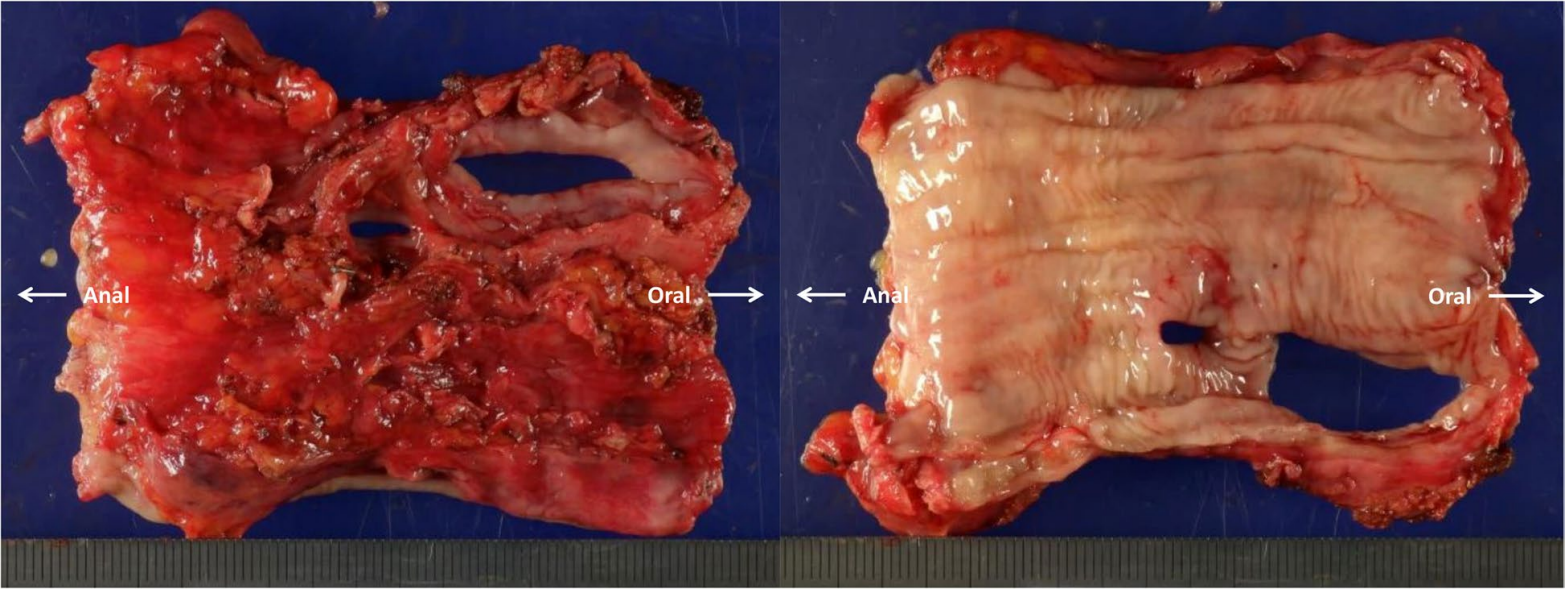
Two holes in the esophagus

The upper body was placed in the left lateral decubitus position, and the lower body was placed in the left hemilateral decubitus position. The surgical approach was a right posterolateral thoracotomy in the 5th intercostal space, and the surgical field was expanded by resecting the 5th and 6th ribs posteriorly. The pericardium was adhered all around. The esophagus was transected at the diaphragm and tracheal carina levels using a Medtronic Signia (60 mm), but it was left in situ until the atrial bleeding was stopped. Heparin was administered, and activated clotting time was > 400 s. An arterial line was inserted into the right common femoral artery and venous line into the right common femoral vein. The esophagus was turned over, and 3-0 polypropylene with a large needle was first inserted into the left atrium through the entire thickness and then through the opposite left atrial wall. Three U-shaped sutures of 3-0 polypropylene were placed at the fistula site to stop bleeding from the atrium (Fig. 2A, B). The esophagus was removed while snaring the 3-0 polypropylene sutures while the heart continued beating, with the bypass pump supporting circulation (Fig. 2C). The bleeding from the left atrium was stopped, so those 3-0 polypropylene sutures were ligated. Further continuous sutures with a 3-0 polypropylene were added for secure closure. Once the atrium was repaired, the cardiopulmonary bypass support was finished. There were two holes in the esophagus (Fig. 3). Four drains were placed to clean the repaired site using saline, and omental wrapping was not performed. After chest closure, the patient was placed supine. Cervical esophagostomy and enterostomy were performed. On postoperative day 28, gastric tube reconstruction via the retrosternal route was performed, and the patient was transferred to another hospital for rehabilitation on postoperative day 99. Nine months after surgery, he visited our hospital for follow-up.

## Discussion and conclusions

Previous studies reported the approaches via median sternotomy [3], left thoracotomy [4], and right thoracotomy [5], but the optimal approach remains controversial. If the fistula on the esophageal side cannot be repaired when repairing the left atrium, there is a risk of prolonged infection at the posterior mediastinum. Therefore, for thorough treatment of AEF, one needs a physical position that allows sufficient separation of the esophagus. We discussed this case with an esophageal surgeon and decided to perform a right posterolateral thoracotomy, which allowed full dissection of the esophagus to the proximal and distal ends, and made safe transection of the esophagus possible.

An important point in treating AEF is to control bleeding. In this case, in order to control bleeding and stabilize the hemodynamics, the patient’s lower body was placed in the left hemilateral decubitus position, and cardiopulmonary bypass was established through the right femoral cannulation. In this position, repair under cardiac arrest is also possible, as is minimally invasive cardiac surgery; so right femoral cannulation seems advisable in patients with AEF.

Another important point is to control infection. In this case, we placed some drains to clean the repaired site and did not perform omental wrapping. A previous study reported the effectiveness of omentum in the management of mediastinal infection [6]. Before performing emergency operation, we discussed with a esophageal surgeon. We decided to repair the fistula site and then perform gastric tube reconstruction in two stages. In order to avoid complicating the procedure such as adhension during gastric tube reconstruction, we did not use the omentum. Fortunately, the infection did not persist after surgery, and we were able to perform the gastric tube reconstruction without any technical problems on the postopereative day 28.

In conclusion, we presented a successful case of AEF. It is important to thoroughly discuss with esophageal surgeon how to reach the heart and esophagus, and how to reconstruct the esophagus later.

## Data Availability

Not applicable.

## Abbreviations

AEF: Atrio-esophageal fistula
